# Manual External Skeletal Distraction of Restricting Soft Tissue after Release of Long-Standing Burn Contractures

**DOI:** 10.1055/s-0045-1806745

**Published:** 2025-03-21

**Authors:** Bharatendu Swain, C. Shravya, Shalini Sampreethi

**Affiliations:** 1 Aakar Asha Hospital, Kukatpally, Hyderabad, Telangana India

**Keywords:** postburn contracture, external fixators, soft tissue distraction, manual distraction, joint forces.

## Abstract

**Background:**

Long-standing burn contractures of limb joints are resistant to complete surgical release and have been addressed earlier by serial casting, and more recently, distraction with a distractor, mostly limited to small joints of the hand. This retrospective study of patients with long-standing burn contracture was done to evaluate the efficacy of external skeletal distractors in securing complete release of various affected joints.

**Materials and Methods:**

In our series, complete release was achieved through gradual manual distraction using skeletal uni- and multiplanar frameworks across the affected limbs after incisional release. The resultant wound after complete release was skin grafted or covered with flaps.

**Results:**

Thirty-one limbs in 27 young patients (average age: 19 years) with long-standing contractures (18.5 years) due to burns underwent manual soft tissue distraction in order to achieve complete release (50–95 degrees across various joints). All patients achieved complete release of contractures with minimal complications.

**Conclusion:**

Gradual manual soft tissue distraction through external skeletal distractors of restricting soft tissue (“stretch as you go”) after incisional release in long-standing burn contracture of limb joints achieves complete release without exposure of vital structures.

## Introduction

The loss of function following a burn contracture can be severe, affecting quality of life due to restriction of activities of daily living and socioeconomic exclusion, and can be an aesthetic concern; they lead to adaptive function, which is less efficient. The hand is frequently involved in patients with a burnt surface area of 15% or more. 1 It is one of the three most frequent sites of burn scar contracture. In low-resource areas, access to quality burns care is limited; therefore, long-standing burn contractures are quite frequently seen. Long-standing contractures of the joints of upper and lower limbs following burns that heal secondarily can result in soft tissue changes, which restrict complete surgical release. Traditionally long-standing burn contractures of limb joints have been addressed by serial casting. 2 Soft tissue distraction emerges as a promising therapeutic approach to address the limitations imposed in long-standing contractures due to a variety of causes. Soft tissue distraction releases restricting soft tissues and promotes tissue regeneration, contributing to better patient outcomes.

In long-standing contractures, secondary changes in the muscle tendon unit, neurovascular structures, and joint capsule occur. The differential growth rate of burn scar and adjacent soft tissue, along with progression of contracture, is more rapid in children than in adults. 3

Soft tissue distraction across joints requires angular forces acting on multiple tissues with varied resistance in comparison to bony distraction, which is linear and mainly involves bone. 4

The physiological changes of bony distraction have been well documented and recommendations made for clinical practice with regard to technique and rate of distraction. However, in the case of deep tissues such as muscle-tendon, nerve, blood vessels, and periarticular structures, though physiological changes have been documented in laboratory experiments, firm clinical recommendations of force and rate of distraction are not available. 5 6

The current series of release of long-standing contractures by manual external skeletal distraction across various limb joints after incisional release of the restricting surface scar, due to a variety of causes, has been evaluated for adequacy of release, duration, and frequency of distraction. The physics of forces acting across a joint were studied and implemented in situations of greater resistance to distraction. Undesirable results have also been documented.

In long-standing joint contractures, incisional release beyond a point will result in exposure of tendon or bone or neurovascular injury. Gradual distraction enables complete release without vital tissue exposure, facilitating cover mostly by split-thickness skin grafts.

## Materials and Methods

A retrospective and ongoing study was conducted from November 2013 to December 2023 within the principles of Helsinki guidelines and with the written consent of the patients. Patient particulars (age, gender, and region), duration of the burn contracture, particulars of deformity (joint involved, angle of contracture), and any associated deformity were noted.

All patients with long-standing burn contractures with failure to secure complete incisional release were included in this study. Immune-compromised patients or those with significant comorbidity were excluded from the study.

**Fig. 1:**
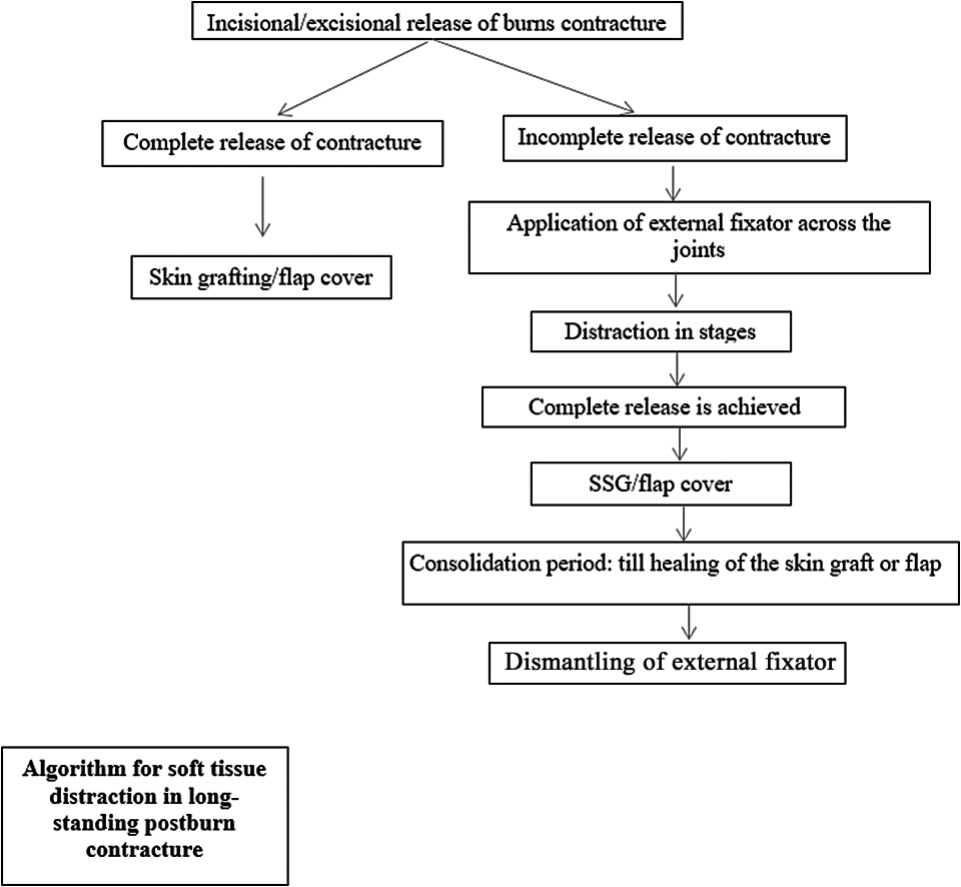
Bidirectional Pathway.

**Fig. 2:**
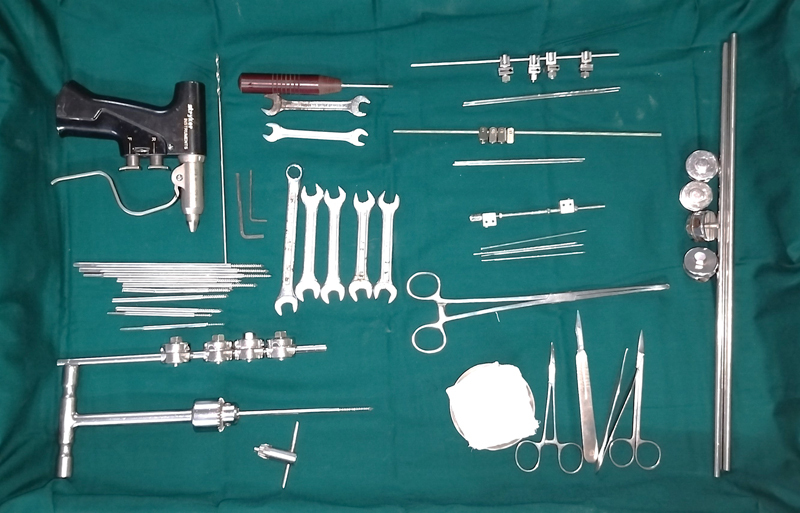
Equipment and instruments for skeletal fixation.

All patients underwent incisional release of the contracture. Where complete intraoperative release was not obtained, external skeletal fixators were applied across the joint and gradual distraction done. Skin grafting was done after complete release was obtained by gradual staged distraction; flaps were done initially in some cases. Consolidation of release was secured for about 2 weeks coinciding with wound healing. Essentially, the consolidation period allowed complete healing in the intended position of release so that the fixation could be removed and physical therapy and splintage commenced soon after. If there was hypergranulation due to delay in securing complete release, it was shaved down before skin grafting. For distraction, a linear external fixator frame spanning the joint was applied using holding skeletal pins of various sizes. Multiaxial distraction was applied where multivectoral release in a region was required. Ilizarov's or Joshi's external stabilization system (JESS) fixators were not used. Manual distraction of the frame was done within the limits of the patient's pain threshold in case of adults and gentle traction in children under sedation. As regards the fixation, a variety of pins (Kirschner wires and Schanz pins), rods, and clamps ( Figs. 1 and 2 ) were used to provide linear or multiaxial fixation or sometimes multihinged fixation.

**Fig. 3:**
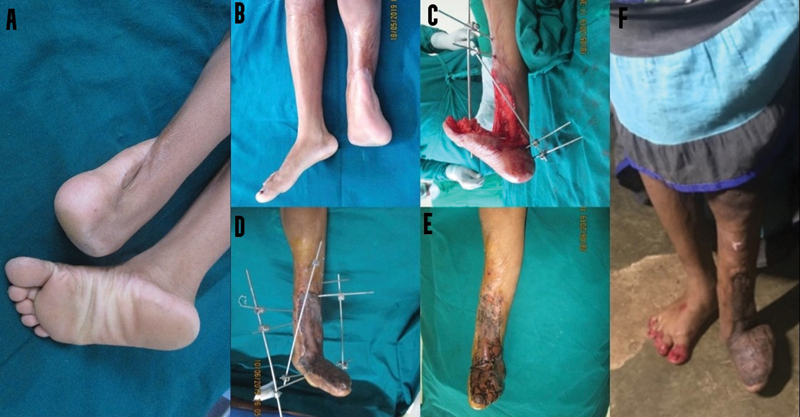
( **A, B** ) Preoperative image. ( **C** ) Release of contracture and external fixator applied. ( **D** ) Skin grafting done after the completion of distraction. ( **E** ) Early postoperative after removal of fixator. ( **F** ) Postoperative review at 6 months.

Additional pins were applied, pins replaced, rod orientation changed, and additional linear framework placed based on the requirements during angular distraction.

Joint angles were measured before surgery, with each distraction and at the time of removal of the distractor apparatus by using a goniometer. Active range of motion and passive range of motion measurements taken were not considered in this study due to multiple adjacent joint involvement or extensive scarring, which deterred accurate measurements.

The distraction rate was determined by tissue resistance, especially for larger joints. Additional pins were added depending on the resistance. The frequency of distraction was planned a week apart, the actual frequency depending on patient's availability and tissue resistance.

After the initial pin breakage in one case of knee contracture, the physics of forces were studied to modify our technique in subsequent three cases of knee contracture, two cases of elbow contracture, and four cases of wrist contracture. The modifications included increasing the number and size of pins across the joints.

In the early postoperative period, active contraction of the antagonist muscles was encouraged to ensure reciprocal relaxation of the agonists in order to facilitate stretching of muscles. Range of motion exercises after removal of pins were initiated, followed by splintage.

Pain (visual analog scale) and complications, if any, were noted. Patients were splinted after removal of the distraction apparatus for at least 6 months; follow-up visits at bimonthly intervals were advised for each patient. After the removal of the distraction frame, patients underwent active and passive mobilization with muscle strengthening by a physical therapist and home therapy afterward for at least 6 months.

In case of patients living at a remote location, a video review was sought.

## Results

Demographic details of 27 patients with 31 affected limbs, which underwent manual soft tissue distraction, were reviewed as shown in Table 1 . The mean age of the patients was 19.1 years (standard deviation [SD]: 11.5; range: 6–65 years), with a male predominance (16:11). The mean time from injury to surgery was 18.5 years (range: 5–29 years; SD: 7.31; Table 1 ). The average gain in release during mid-distraction ranged from 40.2 to 55 degrees (average of 48.9 degrees). The gain during the final range of distraction ranged from 14.5 to 30 degrees (average of 20.08 degrees; SD: 55.1 degrees; Table 2 ). The procedure for initial release and wound closure and associated procedures are listed in Table 3 . The duration of soft tissue distraction by an external fixator was an average of 45 days (range: 6–63 days; SD: 13.8) and the mean duration of the consolidation period after distraction was 15.3 days (range: 6–21 days; SD: 4.2). The number of distractions ranged from one to six times with an average of four times per patient episode (SD: 1.1; Table 4 ).

**Table 1:** Demographic data

Parameters		No. of patients
Age (y)	5–15	6
	16–35	20
	35–65	1
Region	Urban	2
	Rural	8
	Tribal	17
Duration of injury to number of distraction (y)	0–3	3 (3)
	3–7	5 (3)
	7–10	7 (7.45)
	> 10	12(4.9)

Note: The number in brackets denotes the average number of distraction.

**Table 2:** Surgical data

Surgical data		No. of patients	No. of pins used		Rate of distraction		Distraction gain (degrees)	
			Unilateral	Multiaxial	Average days	Average correction achieved (degrees)	Mid distraction	Final distraction
Upper limb	Elbow	4	4		40	65.5	55	20.5
	Wrist	7	4	2	30	55.3	45.6	15.5
	Fingers	6	4	8 (1), 6 (1)	20	61.5	50	20
Lower limb	Knee	6	4 (5), 6 (1)		35	95.6	55	14.5
	Ankle	4	4	6	40	57.3	48	20
	Foot	4	4		30	50	40.2	30

Abbreviation: EF, external fixator.

Note: Bracketed figure denotes number of patients.

**Table 3:** Surgical and associated procedures

Surgical data		No. of patients	Regions (limbs)
Surgical procedure	Incisional release + EF followed by skin grafting	20	Wrist: 6 Elbow: 4 Knee: 5 Ankle: 2 Foot: 3
	Incisional release as a bridge flap + EF followed by skin graft	4	Fingers: 3 Ankle: 1
	Release with 5-flap z-plasty followed by EF	3	Knee: 1 Wrist: 1 Fingers: 1
Associated surgical procedures	Free tissue transfers	2	Ankle: 1 Foot: 1
	Abdominal flap	1	Fingers: 1
	Median nerve graft	1	Wrist: 1

Abbreviation: EF, external fixation.

**Table 4:** Distraction data

	Minimum	Maximum	Average
No. of distractions	1 time	6 times	4 times
Duration of distraction	6 d	63 d	45 d
Consolidation	6 d	21 d	15.3 d
Correction achieved	35 degrees	165 degrees	80.5 degrees

Four case examples are given below, where manual distraction was applied across different joints after standard release was found to be inadequate.

**Fig. 4:**
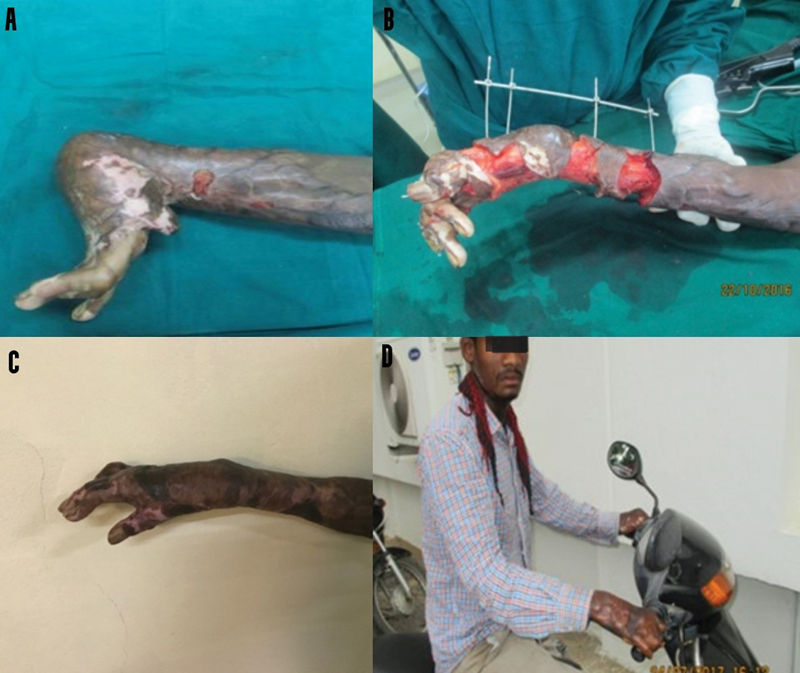
( **A** ) Preoperative image of the right wrist. ( **B** ) Release of contracture and application of external fixator. ( **C** ) Immediately postoperative after the removal of external distractor. ( **D** ) Patient image during the 9-month review.

**Fig. 5:**
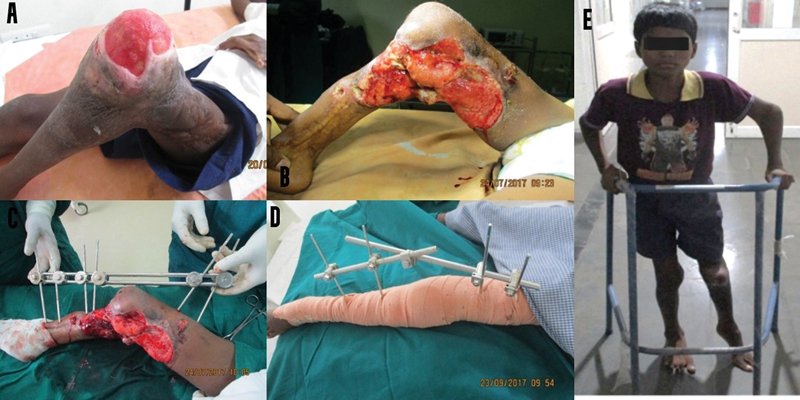
( **A** ) Preoperative image of the left knee. ( **B** ) Release of contracture of the knee. ( **C** ) Application of a fixator. ( **D** ) Postoperative review. ( **E** ) Patient image during the 2-month review.

**Fig. 6:**
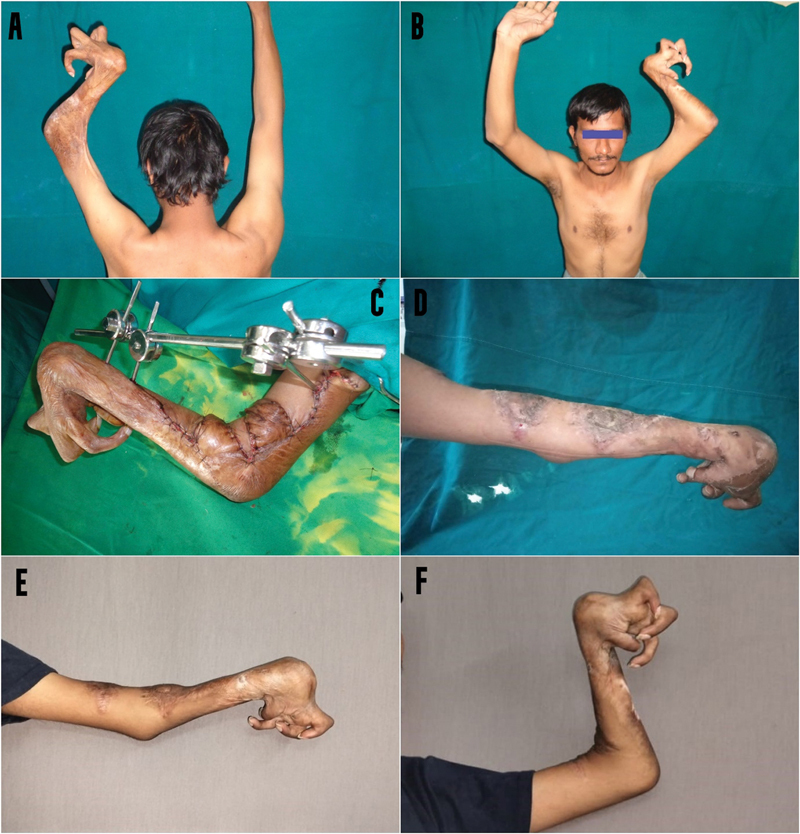
( **A, B** ) Preoperative image of the left elbow. ( **C** ) Release of contracture five-flap z-plasty and application of an external fixator. ( **D** ) Immediate postoperative after removal of external fixator. ( **E, F** ) Patient image at the 1-year postoperative review.

- **Case 1:** A 19-year-old lady sustained domestic burns involving her left foot and leg in early childhood resulting in a severe ankle contracture with the foot buried in the leg; she was walking on the lower end of the tibia. Incisional release could only result in 45 degrees of plantigrade motion. Hence, a multiplanar external fixator was applied across the ankle joint, and distraction commenced to produce plantigrade flexion or correction of everted calcaneum bone. Four distractions were applied over 40 days to secure a 90-degree release to a normal position. The position was maintained for 2 weeks before dismantling the fixator. On review 6 months later, she was found to be able to take steps and walk briskly on her heel ( Fig. 3 ).
- **Case 2:** A 24-year-old tribal man fell into the fire and sustained 40% burns resulting in severe contractures of his neck and both hands. After release of his neck contracture, his right hand contractures were released, and external distraction was done. He underwent extensive physical rehabilitation, which enabled him to get a job as a delivery boy riding a two-wheeler ( Fig. 4 ).
- **Case 3:** A 12-year-old boy sustained burns as a 1-year-old resulting in a flexion contracture of the left knee joint. As he could not walk, he crawled on the floor and unstable scars around the knee led to recurrent ulceration. He underwent wound debridement and release of knee contracture aided by external skeletal distraction. Midway through distraction, the pins had to be re-sited. He achieved complete release and was able to walk with support due to the shorter left lower limb ( Fig. 5 ).
- **Case 4:** A 29-year-old man sustained burns when he fell into open fire as a 2-year-old resulting in severe elbow, wrist, and finger contractures on the left side. He had undergone an unsuccessful release of his left elbow contracture 3 years ago. He was taken up for release of the left elbow contracture with a five-flap z-plasty and external skeletal distraction. In the release of contractures of the upper limb, the sequence of release of proximal structures first is followed to facilitate distal placement of the hand. He achieved complete release but developed 20 degrees of recurrence on review 1 year later ( Fig. 6 ).

## Discussion

Burn contractures not yielding to complete release after scar incision have been traditionally treated by serial casting. 2 The disadvantages of serial casting lie in pressure-induced ulceration of the scars around the joints with the inability to visualize or treat them as they happen. The Illizarov ring fixator was used for distracting a wrist contracture 2 weeks after an unsuccessful initial release. 7 Gulati et al published a large series of distraction of small joints of the hand after release of burn contractures. 8 Illizarov first popularized distraction osteogenesis in the 1970s. He studied the extremities of sheep and dogs for various time periods to determine changes in soft tissues during regenerative changes in bone. He stated that in the presence of tension forces, various amounts of regeneration can occur in muscles and peripheral tissues. He also stated that active formation of myofibrils and sarcomeres takes place in new muscle cells. 9 10

Studies of histologic changes in muscle distraction state that most of the regeneration occurs at the myotendinous junction. Initial stretching is due to easing out of the actin–myosin complex overlap; it is followed by regeneration on sustained distraction. Reorganization of collagen and neohistogenesis are the two mechanisms involved in response to distraction in most tissues including skin, fascia, and periarticular tissue. 5 6 New Schwann cell production and active myelination are seen in stretched peripheral nerves. Owing to sliding of vessels within their surrounding tissue, the traction at first leads only to a straightening of curved blood vessels during distraction, the high viscoelastic properties of blood vessels enabling them to be lengthened considerably without suffering structural damage. Thus, vascular tissue is the least limiting factor concerning distraction speed and rhythm. However, narrow distal arteries traveling through scar tissue can undergo intimal damage and spasm on rapid stretching. 10 11 12

Based on the principle of slow distraction, JESS was developed in the early 1980s. Bony fixation, positioning, and immobilization evolved to ensure stability. 8 13 14

In this study, the surface scar was released by incision, thus limiting the need for distraction to the soft tissues traversing the involved joint(s). The final dimensions of the raw area after release were clearly defined after the last distraction and hence skin grafting was done at the last distraction. In some cases where the release was near complete, with pending one to two distractions, skin grafting was done. Skin grafting of a much smaller raw area on initial release in comparison with the area after complete release will fall short of the complete requirements of resurfacing and will lay the ground for subsequent recurrence of the contracture. In the cases where the distraction period was long, we have shaved granulations (scar precursor) before applying the skin graft.

However, several authors have done distraction a week or 10 days after skin grafting. 8 15

As manual distraction was done in a “stretch as you go” manner, the rate was determined by tissue resistance and pain threshold in adults. The average distraction rate was 9.25 days since most patients, coming from distant places, would attend beyond scheduled review dates as daycare patients for the distractions. However, during the inpatient period, distraction would be done between 6 and 7 days depending on the restricting tissue yield.

There was a positive correlation between the injury–intervention interval and the number of distractions required, across all joints, that is, long-standing contractures required a higher number of distraction episodes ( Table 1 ).

The majority of the patients in this series suffered from long-standing, neglected burn contractures as they came from underserved tribal and rural areas with significant restriction of function. There were some more interesting correlations; comparatively, the wrist and the fingers achieved the most extension (99 and 97%, respectively) and least results were obtained for the elbow and the knee (92 and 90%, respectively). The duration of distraction was the least (average: 16 days) for patients with contracture durations of less than 2 years and the most (average: 41days) for those with contracture durations of more than 20 years. The least number of distractions (average: 2.5) were required when the duration of the contracture was less than 2 years and the highest number of distractions (average: 3.7) in contractures between 2 and 5 years. Most of the cases required uniaxial fixation; of 31 contracture release episodes, 22 underwent unilateral fixation and only 9 underwent multiaxial fixation: 3 ankle, 2 each of the wrist and fingers, and 2 feet.

Manual distraction achieved normal or-near normal extension of all the joints. Regarding surgical procedure and outcome, the results were agnostic with regard to the type of surgical procedures.

In one early case of release of knee contracture, in the terminal phase of distraction, one pin broke and the adjacent pin was bent, underlying the magnitude of forces applied across the joint and the need to distribute the force across a greater number of pins of bigger size and increased pitch of the Schanz pins. In the same case, in the closing stage, neuropraxia of the tibial nerve was produced, which resolved in 2 weeks' time.

While examining the forces applied across the joint, the theta factor (sine of the perpendicular of the joint angle) governs the dilution of force applied as the angle approaches 0 or 180 degrees and maximal retention of force applied as the angle nears 90 degrees. Table 2 clearly shows that the degree of release gained is almost twice in the mid-range than nearing complete release, that is, 0-degree flexion. The closer the frame is to the bone, the more stable it is. An increase in the diameter of the rod increases stiffness and strength. To increase the stability of the bone–pin interface, adequate number of pins in each fragment, increase in pin pitch, and increase in pin size play a major role. 16

The greatest advantage of gradual manual distraction is avoidance of exposure of the vital structures and that it obviates the need for release of the volar plate or collateral ligaments. However, if undue haste is applied resulting in exposure of the vital structures, or in case of correction of the hyperextension of the deformity of the metacarpophalangeal joints, the dermal template would be a very good solution. In this case series, we have not had the need to deal with exposed vital structures.

This case series shows that manual distraction following incisional release of long-standing contractures is effective and safe. The study examines the different aspects of the forces applied across the joint and its application in the clinical context. The later modifications in technique keeping in mind the physics of forces applied 16 improved the ease of release in subsequent cases.

The novelty in this series is the adoption of the external skeletal traction in a “stretch as you go” approach, offering economical and effective treatment, use of readily available hardware, making it versatile, and application of the physics of forces in improving the efficiency of distraction.

The limitation of this study is that as this is an ongoing study, a greater number of cases are planned with long-term follow-up.
